# Clinical characteristics and location of lesions in patients with deep infiltrating endometriosis using the revised Enzian classification

**DOI:** 10.4274/jtgga.galenos.2018.2018.0120

**Published:** 2019-08-28

**Authors:** Fred Morgan-Ortiz, Manuel Antonio López-de la Torre, Marco Antonio López-Zepeda, Fred Valentín Morgan-Ruiz, José Cándido Ortiz-Bojórquez, Martín Adrián Bolívar-Rodríguez

**Affiliations:** 1 Department of Obstetrics and Gynecology, Civil Hospital of Culiacán, Center for Research and Training in Health Sciences, Autonomous University of Sinaloa, Culiacán, Sinaloa, Mexico; 2 Center of Excellence in Endometriosis, San Javier Hospital, Guadalajara, Mexico; 3 Department of General Surgery, Civil Hospital of Culiacán, Center for Research and Training in Health Sciences, Autonomous University of Sinaloa, Culiacán, Sinaloa, Mexico

**Keywords:** Endometriosis, clinical characteristics, surgical findings, deeply infiltrating endometriosis, Enzian classification

## Abstract

**Objective:**

To describe the clinical characteristics and location of lesions in patients with deeply infiltrating endometriosis using the revised Enzian (rEnzian) classification.

**Material and Methods:**

The clinical records of 60 patients undergoing laparoscopy for deeply infiltrating endometriosis at Hospital Civil de Culiacán, Sinaloa and Hospital San Javier, Jalisco, Mexico, were reviewed. Age, body mass index (BMI), number of pregnancies, childbearing, previous abortions, laparoscopic suggestion (pelvic pain, bleeding, infertility), and size and location of the lesions were assessed according to the rEnzian classification.

**Results:**

The mean age of the patients was 30.5 years. The mean BMI was 25.6 kg/m2. Sixty-eight percent were nulliparous and 13% had at least one birth. Eighty-five percent had pelvic pain and 8.3% had infertility. Seventy percent (n=42) of the women had ovarian endometriomas (middle compartment); uterosacral and the torus uterinus ligaments were affected in 23.3%, rectum and sigmoid colon in 35% (posterior compartment), and the appendix and small intestine in 3.3%. According to the rEnzian classification, the most affected compartment was C2 (rectum and sigmoid colon with 1-3 cm lesions).

**Conclusion:**

Pelvic pain was the main symptom of patients with deeply infiltrating endometriosis, mainly in nulliparous women. According to the rEnzian classification, the C2 compartment was the most affected (rectum and sigmoid colon).

## Introduction

Endometriosis is one of the main causes of pain and infertility in women. It can be classified as peritoneal, ovarian, and deep, and affects mostly reproductive-age women (25-35 years), with a rate of 10-15% (1). It is unusual in pre or postmenarcheal women and rare in postmenopausal women (2,3).

The main symptoms reported by patients who are diagnosed as having endometriosis are dysmenorrhea (79%), pelvic pain (69%), dyspareunia (45%), modified gut transit (constipation, diarrhea in 36%), intestinal pain (29%), infertility (26%), ovarian mass (20%), dysuria (10%), and other urinary disorders (6%) (4,5).

Different classifications for endometriosis staging have been proposed based on anatomic location and disease severity. The American Society for Reproductive Medicine (ASRM) score is the most commonly used; it is easily applied and understood by physicians and patients and classifies disease severity in stages I to IV. Among its disadvantages are that staging is not fully correlated with morphologic affection of organs, poor prediction of pregnancy success after treatment, limited reproducibility, and neither retroperitoneal affection nor deeply infiltrating endometriosis are included. Moreover, pain and infertility are poorly correlated with the duration of the disease (6,7).

For this reason, in Austria in 2005, a working group meeting was held with the purpose of forming new classification that included retroperitoneal affection, mainly deeply infiltrating endometriosis (DIE), which was finally designated the Enzian classification (8). However, it is currently not well-known and has a poor level of international acceptance, it is mainly used in German-speaking countries. This first Enzian classification was difficult to use and included anterior, medial, and posterior compartments (8). Then, in 2011, a review of this classification clarified the findings, combining morphologic structures in compartments as with its predecessor, but only considering the posterior portion of the uterus as compartment A (rectovaginal septum and vagina), B (sacrouterine ligaments and pelvic wall), and C (sigmoid colon and rectum), and set the severity of the lesions according to their size as: grade 1 (invasion <1 cm), grade 2 (invasion 1-3 cm) and grade 3 (invasion >3 cm) (9). Invasion to other organs of the lesser pelvis and distance are also considered in this new classification as FA for adenomyosis, FB for bladder involvement, FU for intrinsic ureter involvement, FI for intestinal involvement, and FO for involvement of other organs or structures, such as the abdominal wall. This reviewed version of the classification (2011) was more feasible, useful, and easy to understand by physicians (9).

Several studies have evaluated Enzian classification in relation to its correlation with clinical symptoms and the rASRM classification, reporting that the Enzian classification was partially related to clinical symptoms and severity grades, but significantly correlated with pain and dysmenorrhea, thus, it could be recommended as a complement to the rASRM classification in order to better morphologically describe DIE lesions, even though it requires improvement (10,11,12).

The aim of the present study was to describe the clinical and sociodemographic characteristics, as well as the distribution of lesions according to the revised Enzian (rEnzian) classification in patients with deeply infiltrating endometriosis as observed during laparoscopy.

## Material and Methods

Previously approved by the Ethics and Research Committee, an observational, descriptive, and retrospective study was conducted in patients who were diagnosed and treated for DIE with histopathologic study. Sixty clinical records of patients undergoing laparoscopic surgery from Hospital Civil de Culiacán, Culiacán, Sinaloa and Clínica de Excelencia en Endometriosis, Hospital San Javier, Guadalajara, Jalisco, Mexico, were assessed from July 2010 to July 2016. All patients were diagnosed as having DIE before surgical treatment by a multidisciplinary team that included a gynecologist, coloproctologist, urologist, psychologist, and experts in ultrasound and magnetic resonance imaging.

The Enzian classification (2005) and rEnzian classification (2011) were used to assess the disease (12). The latter version only evaluates DIE location, mainly in the posterior portion of the uterus as described previously.

Analyzed variables were as follows: age, body mass index (BMI), number of pregnancies, childbearing, previous abortions, laparoscopic suggestion (pelvic pain, bleeding or infertility) as well as medical sessions previous to the diagnostic of endometriosis. Surgical findings included number and location of the lesions: anterior compartment (bladder and vesical peritoneum), medium compartment (uterus and ovaries), posterior compartment (rectovaginal septum, uterosacral ligaments (USL), and rectum sigmoid colon) and other locations (e.g. ureter, small gut, appendix), as well as their size. In addition, a description of surgical findings in patients with DIE was reported according to the rEnzian classification related to the distribution and severity of the lesions in compartments A, B, C, FA, FB, FI, and FO.

Statistical analyses included mean and standard deviations for numeric variables, and frequencies and percentages for categorical variables. In addition, 95% confidence intervals (CI) were calculated for each estimate. The SPSS statistical package version 22.0 was used for statistical analyses.

## Results

Mean age of the patients was 30.5 years (95% CI: 28.6-32.3). The mean BMI was 25.7 kg/m2SC (95% CI: 24.8-26.5). The mean number of medical sessions prior to the diagnosis of endometriosis was 7 (95% CI: 5.9-8.0). Regarding the gyneco-obstetric characteristics, 68% of the women were never-pregnant (95% CI: 55.0-79.7), with at least one childbirth 13% (95% CI: 5.9-24.5), at least one cesarean 18% (n=11/60; 95% CI: 9.52-30.43), and at least one abortion 13% (n=8/60; 95% CI: 5.9-24.5). The first symptom to proceed with a diagnostic/surgical laparoscopic procedure was pain in 85% (95% CI: 73.4-92.9), infertility in 8.3% (95% CI: 2.7-18.3), and abnormal genital bleeding in 6.7% (95% CI: 1.8-16.1) (Table 1).

DIE lesions were very commonly found in the medial compartment, in 80% of the subjects (95% CI: 69.5-90.4) (Table 2).

Analysis of DIE lesions by compartment were found in the anterior portion and commonly in the vesical floor (6.6%), with 1-3 cm size in 3.3%, and more than 3 cm in 3.3%. In the medial compartment, the most affected organ was the ovary in 70% (95% CI: 58.4-81.5). The right ovary was the most influenced in 26.6% of the women (95% CI: 16.1-39.6). The size of the lesions most commonly found in this compartment were larger than 3 cm in 45% (95% CI: 32.4-57.6). Related to the posterior compartment, DIE lesions were more frequent in the rectum and sigmoid colon (35%; 95% CI: 22.9-47.1), the most common lesions being 1-3 cm (33.3%; 95% CI: 21.4-45.2). Other unusual lesions were found in the bowel, appendix, and abdominal wall (Table 3).

In regard to the distribution and severity of the lesions according to the rEnzian classification, which does not consider ovaries affection; type C2 (affection to rectum and sigmoid colon with 1-3 cm lesions) was the most commonly found in 23.3%, followed by type B3 (uterus sacral ligaments with lesions larger than 3 cm) in 10% (Table 4).

## Discussion

Infiltrating lesions from DIE are defined as solid focused lesions that invade 5 mm deep or more of organ serosa (13). Some reports indicate that 95% of the lesions involve serosa and muscularis propria, only 38% affect the submucosa and 6% affect the mucosa (14).

DIE is a usual cause of chronic pelvic pain in reproductive-age women. In general, it is associated with anatomic location and the invasion degree of the lesions (>5 mm), (15,16,17) which agrees with the findings in this case series where chronic pelvic pain was the most frequent indication for surgery.

Many endometriosis symptoms are masked by other medical conditions, delaying diagnosis for about 5-10 years when patients have had, on average, 7 medical sessions without a correct diagnosis due to disease unawareness from the first contact with a physician and the patients themselves, who consider the symptoms as normal (18).

The importance of a classification to describe a disease relies on understanding its limits, using the same language when reporting the clinical entity, and reproducing the study within the same terms.

In this trial of 60 cases using the Enzian Classification (2005), the medial compartment was found as the most affected area in 80% of the cases (mainly ovarian endometriomas), followed by the posterior compartment in 65% (mainly rectum and sigmoid colon), and less frequently, the anterior compartment (vesical affection).

Related to the anatomic distribution of endometriosis lesions and a probable physiopathogenic implication, a study revealed that the most affected compartment was the posterior compartment (93.4%), and mainly the left side (67.8%); less frequently, the anterior compartment with vesical affection (6%) (13). This vesical affection report (anterior compartment) is in agreement with the findings in our series of 60 cases where 6.6% was shown; nevertheless, it differs with other reported studies in which 85% were in the bladder, 10% in the ureter, and 4% were found in kidney lesions (19).

Therefore, it could be concluded that DIE is an entity that affects the female pelvis asymmetrically, being more common in the posterior portion and left side of the uterus. This might be explained by the presence of the rectum and sigmoid colon in that side of the pelvis, modifying peritoneal flux in both hemipelvis, thus, blood drops retrogradely during menstruation and accumulates in this area of the pelvis, leading to implantation of endometrial cells and disease development (13).

The presence of endometriomas (medial compartment) could be a marker of endometriosis severity, mainly DIE. In the present series of 60 cases with DIE, 70% of the patients showed an endometrioma more often on the right than on the left side, in disagreement with a previous study hypothesis proposing anatomic distribution of the pelvis. This frequency is similar to 77% of endometriomas in patients with DIE (rectum and sigmoid colon involvement) compared with 21% without endometrioma (risk ratio: 6.96; 95% CI: 4.04-12.00) (20).

It is important to mention that ovarian endometriosis is a marker of spread pelvic disease, and associated with cul-de-sac obliteration involving the rectum, sigmoid colon, and the seromuscular layer of the bowel, which should be treated if surgery favors the patient, even when ovarian affection absence does not discard DIE as a possibility (21). Moreover, USL affection could be a marker of ureteral involvement by DIE (22,23). In a study with 463 patients DIE with presurgical transvaginal ultrasound, 111 patients showed USL involvement. Ureter affection was associated with ovarian mobility, ureteral changes on the right side, nodule size of USLs, and endometrioma on the left side, particularly when USLs were 1.75-1.95 cm, in the right and left sides, respectively (22).

The Enzian classification in 2005 was poorly accepted due to its complex clinical application, so it was revised and modified in 2011 (rEnzian) (8,9). This 2005 review only included affection of endometriosis from the posterior compartment. In the revised classification, posterior compartment of the uterus was divided in three as A, B, C and F, and severity goes with nodule size (G1, G2, and G3) in such a way that tumor, node, metastasis staging could be used as in malignant diseases; therefore, a presurgical description of involved organs using the compartment and severity of the lesion is possible. For example, a presurgical patient with DIE using the Enzian classification would be A0 B1 C2 F (with no lesions in the rectovaginal septum and vagina, with less than 1 cm lesions in the USLs and pelvic wall, and 1-3 cm lesions in the sigmoid colon).

In the present case series, the most affected compartment was C with 21 cases, 14 of which were grade 2. Thus, to describe this lesion it should be classified as C2, which means that the most affected sites in the patients of this trial were the rectum and sigmoid colon with infiltrative lesions from 1-3 cm. This implies that patients would require a discoid or segmental resection of those organs, the surgeon should then anticipate instrumental provisions, surgical time and, most importantly, a multidisciplinary team to continue the procedure. In Mexico and other countries around the world, there is little knowledge of DIE treatment as a multidisciplinary disease, where imaging experts and surgeons work together with a close communication to handle patients with DIE.

One of the biggest problems for physicians is having presurgical diagnostic confidence of the disease relying on cost/benefit and less invasive techniques such as ultrasound, which in skilled experts has an excellent sensitivity and specificity to diagnose DIE, even similar to magnetic resonance (24,25). Unfortunately, most centers in Mexico lack trained personnel to diagnose DIE because they have never faced this problem in the past or do not know about its existence.

The same happens with DIE surgical treatment as a multidisciplinary entity; few groups at national level work on integral DIE treatment; however, with that goal, an accurate diagnostic of involved organs is required in order to anticipate the needs for a correct management, as mentioned.

Accordingly, the rEnzian classification becomes useful as in the present study where, even with a small sample, the frequency of affected organs was described clearly and could simplify pre and post-surgical reports.

**Synopsis:** Deeply infiltrating endometriosis occurs mainly in young women with pelvic pain and lesions that are often located in the C2 compartment according to the rEnzian classification.

## Figures and Tables

**Table 1:**
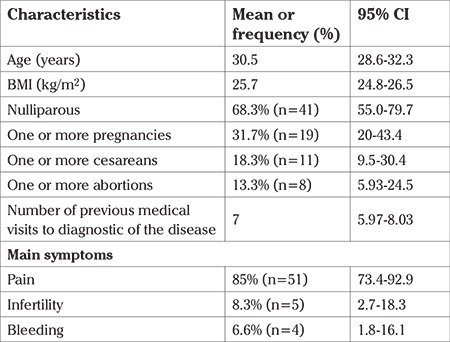
General characteristics of the studied population

**Table 2:**
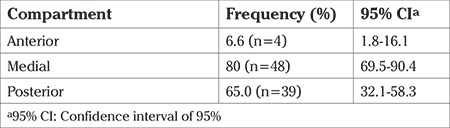
Location of deep infiltrating lesions by compartment

**Table 3:**
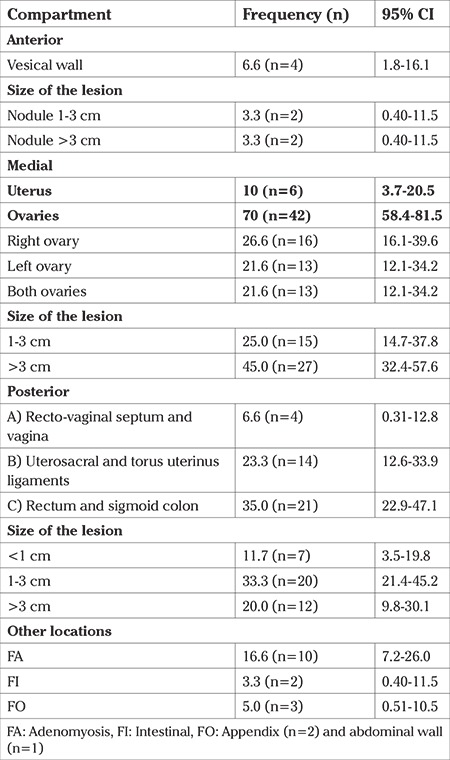
Distribution and size of deep infiltrating lesions by compartment

**Table 4:**
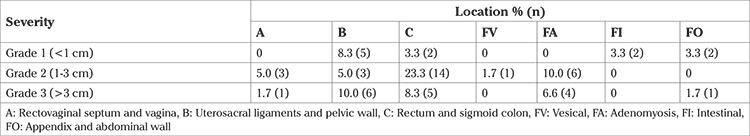
Distribution and severity of deeply infiltrating endometriosis in agreement with the revised Enzian classification (2011)

## References

[1] Wang G, Tokushige N, Markham R, Fraser IS (2009). Rich innervation of deep infiltrating endometriosis. Hum Reprod..

[2] Sangi-Haghpeykar H, Poindexter AN (1995). Epidemiology of endometriosis among parous women. Obstet Gynecol.

[3] Steele RW, Dmowski WP, Marmer DJ (1984). Immunologic Aspects of Human Endometriosis. Am J Reprod Immunol.

[4] Sinaii N, Plumb K, Cotton L, Lambert A, Kennedy S, Zondervan K, et al (2008). Differences in characteristics among 1,000 women with endometriosis based on extent of disease. Fertil Steril.

[5] Dunselman GA, Vermeulen N, Becker C, Calhaz-Jorge C, D'Hooghe T, De Bie B, et al (2014). ESHRE guideline: management of women with endometriosis. Hum Reprod.

[6] No authors listed (1997). Revised American Society for Reproductive Medicine classification of endometriosis: 1996. Fertil Steril.

[7] Haas D, Shebl O, Shamiyeh A, Oppelt P (2013). The rASRM score and the Enzian classification for endometriosis: their strengths and weaknesses. Acta Obstet Gynecol Scand.

[8] Tuttlies F, Keckstein J, Ulrich U, Possover M, Schweppe KW, Wustlich M, et al (2005). ENZIAN-score, a classification of deep infiltrating endometriosis. Zentralbl Gynakol.

[9] No authors listed (2011.). Stiftung Endometriose Forschung. The revised Enzian classification. Consensus meeting, 7th Conference of the Stiftung Endometriose Forschung (SEF) (Foundation for Endometriosis Research), Hotel Enzian, Weissensee, Austria, February 25-27, 2011. Weissensee, Austria.

[10] Haas D, Oppelt P, Shebl O, Shamiyeh A, Schimetta W, Mayer R (2013). Enzian classification: does it correlate with clinical symptoms and the rASRM score?. Acta Obstet Gynecol Scand.

[11] Haas D, Chvatal R, Habelsberger A, Wurm P, Schimetta W, Oppelt P (2011). Comparison of revised American Fertility Society and ENZIAN staging: a critical evaluation of classifications of endometriosis on the basis of our patient population. Fertil Steril.

[12] Haas D, Wurm P, Shamiyeh A, Shebl O, Chvatal R, Oppelt P (2013). Efficacy of the revised Enzian classification: a retrospective analysis. Does the revised Enzian classification solve the problem of duplicate classification in rASRM and Enzian?. Arch Gynecol Obstet.

[13] Chapron C, Chopin N, Borghese B, Foulot H, Dousset B, Vacher-Lavenu MC, et al (2006). Deeply infiltrating endometriosis: pathogenetic implications of the anatomical distribution. Human Reprod.

[14] De Cicco C, Corona R, Schonman R, Mailova K, Ussia A, Koninckx P (2011). Bowel resection for deep endometriosis: a systematic review. BJOG.

[15] Abrao MS, Podgaec S, Dias JA Jr, Averbach M, Silva LF, Marino de Carvalho F (2008). Endometriosis Lesions That Compromise the Rectum Deeper than the Inner Muscularis Layer Have More Than 40% of the Circumference of the Rectum Affected by the Disease. J Minim Invasive Gynecol.

[16] Donnez J, Squifflet J (2004). Laparoscopic excision of deep endometriosis. Obstet Gynecol Clin Nort Am.

[17] Howard FM (1996). The role of laparoscopy in the evaluation of chronic pelvic pain: Pitfalls with a negative laparoscopy. J Am Assoc Gynecol Laparosc.

[18] Ballard K, Lowton K, Wright J (2006). What’s the delay? A qualitative study of women’s experiences of reaching a diagnosis of endometriosis. Fertil Steril.

[19] Yohannes P (2003). Ureteral Endometriosis. J Urol.

[20] Banerjee SK, Ballard KD, Wright JT (2008). Endometriomas as a Marker of Disease Severity. J Minim Invasive Gynecol.

[21] Redwine DB, Wright JT (2001). Laparoscopic treatment of complete obliteration of the cul-de-sac associated with endometriosis: long-term follow-up of en bloc resection. Fertil Steril.

[22] Lima R, Abdalla-Ribeiro H, Nicola AL, Eras A, Lobao A, Ribeiro PA (2017). Endometriosis on the uterosacral ligament: a marker of ureteral involvement. Fertil Steril.

[23] Carfagna P, De Cicco Nardone C, De Cicco Nardone A, Testa AC, Scambia G, Marana R, et al (2017). Role of transvaginal ultrasound in evaluation of ureteral involvement in deep infiltrating endometriosis. Ultrasound Obstet Gynecol.

[24] Turocy JM, Benacerraf BR (2017). Transvaginal sonography in the diagnosis of deep infiltrating endometriosis: A review. J Clin Ultrasound.

[25] Exacoustos C, Lazzeri L, Zupi E (2017). Expert sonographers and surgeons are needed to manage deep infiltrating endometriosis. Ultrasound Obstet Gynecol.

